# The Territorial Regimental Surgeon

**Published:** 1909-01-23

**Authors:** 


### The Royal Army Medical Corps Section.

### I.—THE TERRITORIAL REGIMENTAL SURGEON.

It was on March 1, 1873, that there was introduced into our Regular Army the very radical change of complete abolition of the regimental medical system. After that date all Army medical officers belonged to what was then the Medical Staff Corps or Department; the regimental hospitals ceased to exist, and all the military hospitals were organised and administered as general, station, or field hospitals. It is not necessary to go fully into the circumstances which led to this very material change in the medical organisation of our Regular Army, but it may be stated generally that it was the outcome of the failure of the regimental system to meet the needs of the sick and wounded soldiers in the Crimean War. At first an endeavour was made to supply the deficiencies by forming, as was done in July, 1855, a Medical Staff Corps of nine companies, each of 78 strong, and so arranged "as to be employed in any way that may be required in the performance of hospital duties." The result of this new arrangement was that for some time there worked side by side in the Regular Army two systems, as it were, the regimental one and that of the Medical Staff Corps; the former dealt with the medical needs of the individual regiments in time of peace, the latter was trained and organised to supplement the other in time of war so as to meet the extra demands that would be then made on the medical service. In the course of time the view recommended itself to the military authorities that it would be better to have one single organisation instead of what was practically two, and the abolition of the regimental system in its entirety took place, as already mentioned, in 1873.

If the medical and surgical needs of the Army were alone to be provided for, the change to the "unification system," as it was termed, is decidedly advantageous; but if, as is very properly held in the present day, the prevention of disease is one of the chief uses of an Army medical service, then there is much to be said in favour of the old regimental system. Under it, the medical officer attached to a regiment, if tactful and of good professional ability, exercises a most beneficial influence over officers and men on all matters of health, an influence that is completely lost when he is removed. Looking at the matter now dispassionately there can be no doubt that the breaking up of the regimental medical system, before there was built up a system to take its place, was a mistake disadvantageous to the welfare of the Army in the matter of sanitation. The knowledge we now have of the origin of diseases, especially of those infectious ones that have so often decimated armies in the field, makes it clear that the position of medical officer to any unit furnished a splendid opportunity of communicating to all ranks of the unit recent advances in disease prevention, and of enlisting the sympathies and support of the combatant officers in obtaining a ready obedience to all instructions on sanitary matters.

It is not improbable that recognition of this fact influenced the military authorities in their decision to include a modified regimental system in the medical service of the new Territorial Army. Under the new *régime*, as already explained in a previous number of this Journal, there is to be one consolidated R.A.M.C. (T.F.), to which all medical officers must belong, but those who desire may elect to serve with regiments, the number serving with each regiment being limited to two. The duties of such regimental surgeons are now very definitely laid down, and they are more extensive and more important than those that belonged to the Volunteer regimental medical officer. Briefly, they may be said to consist in giving instruction in: (1) First aid and stretcher drill; (2) sanitation.

As was the case before, regimental medical officers are responsible for the regimental or first line of assistance to the sick and wounded of their unit. To enable them to carry out this duty they are furnished with one corporal and 16 men as stretcher-bearers, all of whom must be instructed in a practical knowledge of first aid and stretcher drill. It is also advisable that they should have some knowledge of nursing duties, for they must in peace time furnish attendants for any sick or injured in the regimental or brigade hospital tents. An idea exists that such attendants will be furnished by the field ambulances doing duty with their respective brigades, but this is not so. These units go to camp simply for combined training and only deal with the sick and wounded on field days and manœuvres, handing them over to the regimental medical authorities immediately on their return to camp. This being the arrangement it is evident that regimental surgeons must rely on their own personnel for any nursing attendants they may require and should instruct them accordingly. As only cases of minor ailments and accidents will be retained in the regimental or brigade hospital tents during camp, no elaborate instruction in nursing duties is required. According to regulations the corporal and 16 men must be taken from the bandsmen of the regiment, but if such men are not available the commanding officer must furnish the necessary quota from the regimental strength. When in camp the regimental medical officer will be furnished with the following medical and surgical equipment, if requisitioned for on the proper Army form—namely, one medical companion and water-bottle, one surgical haversack and water-bottle, one pair field medical panniers, and one fracture box. In the case of mounted troops there is supplied in addition to the above one pair surgical saddle-bags and water-bottle. As at present contemplated, the above equipment will be held on charge all the year round by the different units, and will be available for instructional purposes as well as for camping. It is, however, in the new departure of sanitary instruction that the position of the regimental medical officer has assumed an importance and responsibility that did not attach to it previously. Under the new scheme every regiment must have its sanitary squad and its water-duty men. For the former one non-commissioned officer and eight men are furnished out of the regimental strength. Some commanding officers supply these men out of the ten pioneers that are usually attached to each regiment. No doubt these latter are excellent for a regimental sanitary section, but they are not always available as they have other duties to perform, so that possibly specially enlisted men for the purpose would be better. Whatever, however, be the source from which they are supplied, their training in sanitation falls to the regimental surgeon. So, too, does that of the water-duty men, although it is possible he may be relieved of this last additional work as the view seems to be held that the water-duty men should have their training as a body at the school of instruction of the division to which their regiments belong, and be sent out from there to their respective units on mobilisation or when in camp.

The lines of sanitary instruction that should be followed by the regimental surgeon are those laid down in the R.A.M.C. Training Manual, and include both general and personal hygiene. Thus the sanitation unit of a regiment should be well and practically taught in the best methods of the disposal of refuse and excreta, and to act as sanitary police, while the water-duty men should be specially trained in everything connected with the water supply and the disinfection of the sick, these latter being a frequent source of water contamination. It is, however, in the matter of personal hygiene that the regimental surgeon is such a potent factor for good. No opportunity should be lost of impressing on company officers and men the necessity and importance of their co-operation in this matter, and it should be strengthened by printed rules. The chief points in personal hygiene that should be dwelt on are: (1) The care of the body, even to minor details as the teeth, cleansing of hands, and keeping of hair short; (2) attention to food and drink, excess being avoided, and uncooked food never being partaken of; (3) precaution during marching as to cleansing and filling of water-bottles, protection of the head and care of the feet; and (4) the genesis of infectious diseases by improper food and foul water. By constant and simple instruction on this subject of personal hygiene and by insisting on its being made part of the soldier's daily life, a field of immense usefulness is opened up to the regimental surgeon, for the efficiency of his unit as a fighting force may be said to lie largely in his hands.

